# EFFECT OF TRADITIONAL REHABILITATION PROGRAMME VERSUS TELEREHABILITATION IN ADOLESCENTS WITH IDIOPATHIC SCOLIOSIS DURING THE COVID-19 PANDEMIC: A COHORT STUDY

**DOI:** 10.2340/jrm.v56.5343

**Published:** 2024-02-26

**Authors:** Rodrigo MANTELATTO ANDRADE, Bruna GOMES SANTANA, Ariane VERTTÚ SCHMIDT, Carlos EDUARDO BARSOTTI, Marina PEGORARO BARONI, Bruno TIROTTI SARAGIOTTO, Ana Paula RIBEIRO

**Affiliations:** 1 University of Sao Paulo, School of Medicine, Physical Therapy Department; 2 Clinical Center in Scoliosis, São Paulo; 3 Medicine Department, Health Science Post-Graduate, Biomechanics and Musculoskeletal Rehabilitation Laboratory, University Santo Amaro; 4 Member of the Spine Group, Hospital do Servidor Público Estadual; 5 Masters and Doctoral Programs in Physical Therapy, Universidade Cidade de São Paulo, São Paulo/SP, Brazil

**Keywords:** idiopathic scoliosis, adolescents, exercise, physical therapy, telerehabilitation

## Abstract

**Background:**

Telerehabilitation has become increasingly popular since the SARS-CoV-2 (COVID-19) outbreak. However, studies are needed to understand the effects of remote delivery of spine treatment approaches.

**Objectives:**

To verify and compare the effects of traditional rehabilitation programmes (in-person) and telerehabilitation (online) on the progression of scoliotic curvature in adolescents with idiopathic scoliosis during the COVID-19 pandemic, and to verify the acceptability, appropriateness, and feasibility among patients and physiotherapists regarding both treatments.

**Methods:**

This is a cohort study (prospective analysis of 2 intervention groups: telerehabilitation (online) and traditional rehabilitation (in-person). A total of 66 adolescents with idiopathic scoliosis were included. Recruitment was conducted through the Clinical Center in Scoliosis Care (January–December 2020). Participants were divided into 2 intervention groups: telerehabilitation (online) (*n* = 33) and traditional rehabilitation programme (in-person) (*n* = 33). Both groups also were supplied with a spinal orthopaedic brace. Scoliosis was confirmed by a spine X-ray examination (Cobb angle). Radiographic parameters measured were: Cobb angles (thoracic and lumbar). The method of Nash and Moe (thoracic and lumbar) was also evaluated based on the relationship between the vertebral pedicles and the centre of the vertebral body in the X-rays. Assessments were performed at baseline (T0) and after 6 months of the intervention protocol (T6). Patient and physiotherapist reports were evaluated on the acceptability, appropriateness, and feasibility of the interventions.

**Results:**

Adolescents with idiopathic scoliosis showed a significant decrease in the Cobb angle (main scoliotic curvature), with a 4.9° for the traditional rehabilitation programme and 2.4° for the telerehabilitation. Thoracic and lumbar Cobb angles did not show significant changes after the intervention in both groups or between groups. Thoracic and lumbar Nash and Moe scores scores also did not show significant differences after 6 months of in-person or telerehabilitation intervention, or between groups. The intervention by telerehabilitation was acceptable, appropriate, and feasible for patients and physiotherapists.

**Conclusion:**

Use of the rehabilitation programme for adolescents with idiopathic scoliosis, delivered via telerehabilitation during the COVID-19 pandemic, was encouraging for future applications due to the improved effect on reducing the Cobb angle, preventing progression of scoliosis. In addition, telerehabilitation showed good acceptability among patients and physiotherapists. Traditional rehabilitation programmes (in-person) in adolescents with idiopathic scoliosis also showed a reduction in the Cobb angle.

LAY ABSTRACT

Telerehabilitation has become increasingly popular since the SARS-CoV-2 (COVID-19) outbreak. Telerehabilitation involves virtual patient care and assistance with exercises for patients who have remote or limited access to physiotherapy care. This study compared the effects of a traditional rehabilitation programme (in-person) and telerehabilitation (online) on the progression of scoliotic curvature in adolescents with idiopathic scoliosis during the COVID-19 pandemic, and studied the acceptability, appropriateness, and feasibility among patients and physiotherapists regarding both treatments. Adolescents with idiopathic scoliosis showed reductions in the Cobb angle with both telerehabilitation and the traditional rehabilitation programme. The intervention was acceptable, appropriate, and feasible for patients and physiotherapists. Thus, the effect of the rehabilitation programme, delivered via telerehabilitation during the COVID-19 pandemic, was encouraging for future applications, due to improvement in reducing the Cobb angle, preventing the progression of scoliosis among these patients.

Telerehabilitation involves virtual patient care through telecommunication technology tools (1). This form of patient care can provide interventions with physiotherapy exercises, telehealth with clinical assistance using virtual consultations, and patient monitoring on a digital platform, without the physical presence of a health professional (2). Telerehabilitation can be performed synchronously or asynchronously. Synchronous refers to the delivery of real-time rehabilitation (3), which allows for live supervision and training between the patient and the physiotherapist. Asynchronous telerehabilitation is carried out through sharing recorded videos or exercise images with the patient, without the need for simultaneous or real-time access by the physiotherapist (3, 4).

The advantages of telerehabilitation include assistance with exercises for patients who have remote or limited access to physiotherapy care (5, 6), the possibility of accessing specialized care in a virtual form (7), healthcare monitoring (8), and lower cost of clinical healthcare (9). Telerehabilitation could potentially enable the equitable delivery of health services, particularly as adequate internet access continues to increase globally (10). A wide range of studies have investigated the effectiveness of telerehabilitation in the management of health conditions, with reported results of non-inferiority compared with in-person healthcare (11, 12), and with positive effects on quality of life, and patient satisfaction with the treatment of musculoskeletal disorders (13, 14). A systematic review, in 2023, with 10 repeated-measures studies involving 193 participants aged 23–62 years, showed evidence of the effectiveness of face-to-face physiotherapy assessment (traditional) vs digital assessment of musculoskeletal disorders, concerning validity, reliability, patient and physiotherapist satisfaction, and cost-effectiveness (15).

Among the musculoskeletal disorders, adolescents with idiopathic scoliosis (AIS) require special attention, given the need for assistance, due to the skeletal maturity phase, which can result in progression of curvature by approximately 5.4° to 9.6° over 6 consecutive months (16). Many complications can be associated with the progression of scoliotic curvature, such as pulmonary involvement, chronic pain, vertebral deformity, and psychosocial factors that result in expenditure on health services directed towards conservative or surgical treatment (17, 18).

Current evidence demonstrates the importance of conservative treatment, using specific exercises to minimize the progression of the Cobb angle, given the biomechanical action of AIS on the spine, resulting in an imbalance of forces and postural asymmetry that worsens motor coordination in adolescents (19). Conservative treatment with specific exercises for AIS is based on the recommendations of the International Society on Scoliosis Orthopaedic and Rehabilitation Treatment (SOSORT) and an indication for the use of orthopaedic braces, when necessary (20–22). In general, the recommendation is for scoliotic curvatures between 25° and 45° to be treated with exercise and brace on spine, with highly positive clinical evidence to support progressive reduction and control of the Cobb angle due growth potential of the skeletal maturity phase in adolescents (23).

There is preliminary evidence to suggest that the adoption of telerehabilitation instead of face-to-face interventions is beneficial for reducing pain and improving physical function in patients with chronic non-malignant musculoskeletal pain due to low-back pain, lumbar stenosis, neck pain, and osteoarthritis (11). Telerehabilitation interventions have proven beneficial for patients with low-back pain to reduce pain levels and maintain that improvement via booster sessions delivered through a mobile phone application and videoconferencing (11–13). Under circumstances such as the COVID-19 era, telerehabilitation would enable the delivery of rehabilitation interactions on a larger scale (12, 13). According to Fiani et al. (11), studies have shown that telerehabilitation is well-received by patients as a stand-alone treatment or when supplemented with conventional face-to-face therapy. Although the beneficial effects of conservative treatment with specific exercises for AIS, delivered via in-person rehabilitation, to minimize scoliotic curvature have been verified, no studies were found on the benefits of such treatment applied virtually, i.e. by telerehabilitation.

Variations in the use of telerehabilitation, and a lack of rigorous studies specifically tailored to patients with spinal conditions, limits conclusions regarding whether telerehabilitation services should be used more broadly for mainstream delivery of rehabilitation beyond the current situation of the COVID-19 pandemic. Large, well-powered, long-term studies are required to determine the specific indications for telerehabilitation in spine patients and to explore patient outcomes, which justifies the performance of the current study. It is clear that, with recent advancements in technology, and increasing availability of low-cost platforms for telerehabilitation services, this field will continue to expand in the future. Further research is needed to determine evidence-based methods and the cost-effectiveness of services in order to support the use of telerehabilitation and increase reimbursement by health insurance providers and enable reimbursement where the healthcare system depends on health insurance. Thus, the objectives of this study are to verify and compare the effects of traditional (in-person) rehabilitation programmes with (online) telerehabilitation on the progression of scoliotic curvature in AIS during the COVID-19 pandemic, and to verify the acceptability, appropriateness, and feasibility of both treatments among patients and physiotherapists. This study hypothesized that the effects of telerehabilitation would be similar to those of traditional rehabilitation with regards to reducing and controlling the Cobb angle in AIS.

## MATERIAL AND METHODS

### Study design and participants

This is a cohort study with an implementation perspective and prospective analysis of 2 intervention groups: telerehabilitation (synchronous) and traditional in-person rehabilitation. A total of 66 volunteer AIS were included, who were evaluated pre-intervention (baseline) and post-intervention (6 months after the end of the intervention). The intervention included specific exercises and thoracic-lumbar-sacral orthosis (TLSO), according to SOSORT 2018 guidelines (21), performed via telerehabilitation (synchronous) (*n* = 33) or traditional in-person rehabilitation (*n* = 33). Recruitment was conducted through the Clinical Center in Scoliosis Care in the State of Sao Paulo/SP, Brazil. This study was previously submitted to the Research Ethics Committee of the University of Santo Amaro/SP (approval number 4.943.361). Before participation, all adolescents or their respective legal guardians, signed the free and informed consent form prepared by the Declaration of Helsinki and regulations.

The eligibility criteria were: adolescent, between the ages of 10 and 17 years, a diagnosis of AIS confirmed by X-ray with a Cobb angle between 30° and 45° of the principal curvature (thoracic or lumbar, according to the Lenke classification), and a body mass index (BMI) less than 35 kg/cm^2^. The exclusion criteria were: musculoskeletal disorders in the lower limbs related to the central and peripheral nervous system, diabetic neuropathies, rheumatoid arthritis, rigid foot deformities, previous or planned spinal surgery in the next 12 months, mental health disorder, prostheses and/or orthoses in the lower limbs or fractures in the previous 6 months, and receipt of any other physical therapy treatment during the intervention period.

### Radiographic and clinical evaluations

Assessments were performed at baseline (T0) and 6 months after the end of the intervention period (T6). Full-length, free-standing spine radiographs with fists on clavicles were obtained in all subjects and measured by experienced radiation technologists. The radiographs were centred on T12 during inspiration, with a 2-m distance between the film and the focus. All images were transferred to a computer as digital images and evaluated using the image software Surgimap Spine (Nemaris Inc., New York, USA) (23, 24).

Sagittal alignment parameters were analysed on the radiographs of the 66 participants: Cobb angle of the main curve according to the classification of Lenke, thoracic Cobb angle (T1–T12), and lumbar Cobb angle (L1–S1) (24). The thoracic Cobb angle was measured using the T1 and T12 plateaus, and the lumbar Cobb angle was measured using the angle formed between the upper endplate of L1 and S1. The radiographic evaluations were always performed by the same radiologist, in order to maintain standardization of the X-ray images. The post-rehabilitation treatment images were taken 6 months after the end of the intervention.

The Risser system divides the steps of ossification and fusion of the iliac apophysis into 6 stages (Risser Stages 0–5), with the higher numbers describing advancement toward skeletal maturity (25). Stage 0 describes an X-ray on which no ossification centre is seen in the apophysis, whereas Stage 5 represents complete ossification and fusion of the iliac apophysis. Two slightly different versions of the Risser system are in use; the differences concern Stages 2 to 4. The staging system divides the excursion of the apophysis into quarters of the iliac crest, beginning anterolaterally and progressing posteromedially (25, 26).

The Nash and Moe method of determining vertebral rotation clinically divides the apical vertebral body into 6 equal segments longitudinally. The Nash and Moe score is rated from 0 to 4. When both pedicles are in view, there is no vertebral rotation, and the grade is 0; grade 1 is when the pedicle on the concave side starts to disappear; grade 2 when the pedicle disappears; grade 3 when the contralateral pedicle is in the midline of the vertebra; and grade 4 when it crosses the midline of the vertebra (26).

*Data reliability analysis.* To verify the degree of reliability of the intra-examiner analysis, a single examiner (a doctor experienced in evaluations) measured the sagittal angles and spine parameter (°) with an interval of 1 week between the first and second X-ray assessments to ensure that there was no memorization of the angles.

### Intervention protocol

Participants were divided into 2 intervention groups: telerehabilitation exercises group and in-person exercises group. Both groups were treated with the specific dynamic exercise programme for 6 months. Both groups received supervision from a physiotherapist (25). In both groups, the programme was carried out once a week for 60 min for 6 consecutive months combined with the use of a spinal brace (mean use 18–20 h daily). After each session, the physiotherapist performed an educational process with exercise guidance for weekly practice in the home environment. The brace was monitored daily by the physiotherapist, when contacting the patient by telephone to record the hours of use.

In the traditional in-person group, participants received a specific exercise programme with assistance at the specialized referral centre for AIS care. In the telerehabilitation group, participants received a specific exercise programme using a videoconference platform (WhatsApp video) performed synchronously.

**Fig. 1:**
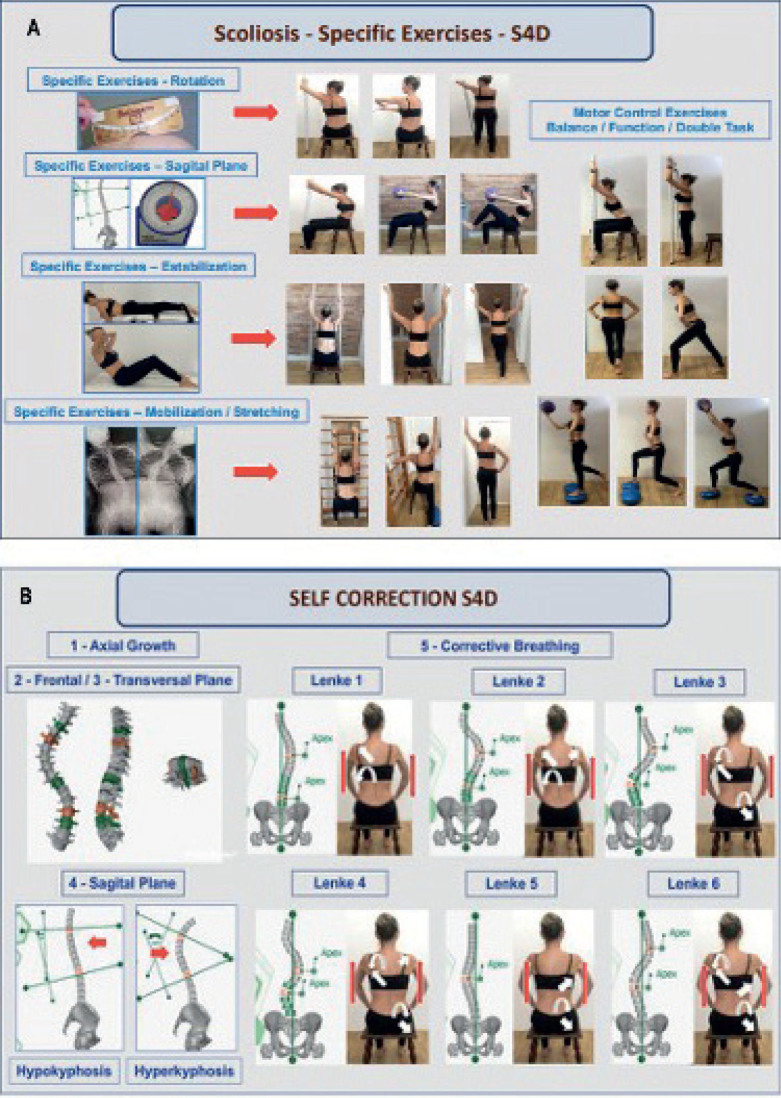
Representation of the intervention programme for patients with adolescents with idiopathic scoliosis (AIS). (A) Training with specific exercises. (B) Training of the self-correction with specific exercises of the programme.

All patients were treated according to a specific exercise programme, prescribed for the scoliotic curvature pattern, based on the Lenke classification. The specific exercise programme was proposed to self-correct the trunk and provide training to maintain the corrected posture while performing activities of daily living (ADL). The intervention programme with specific exercises is described below (see also Fig. 1) (25):

- *Axial growth:* patient seated, with feet and knees apart and aligned forward, hands pushing the thighs for axial growth of the trunk;
- *Frontal plane/vertebral tilt correction:* shoulder elevation on the contralateral side of the scoliotic curve to open the ribs in thoracic/thoracolumbar curves with thoracic emphasis; press the hip on the contralateral side of the scoliotic curve to open the lumbar/thoracolumbar curve with lumbar emphasis;
- *Correction of the transverse plane:* rotational movement of the vertebrae to the opposite side from which the vertebrae are rotated. Perform the movement by rotating the pelvic girdle for lumbar/thoracolumbar curves with lumbar emphasis; perform the movement by rotating the shoulder girdle for thoracic/thoracolumbar curves with thoracic emphasis.
- *Correction/maintenance of the sagittal plane:* for thoracic hypokyphosis, a thoracic flexion movement was performed, keeping the lumbar and cervical spine stabilized. For thoracic hyperkyphosis, a thoracic extension movement was performed, keeping the lumbar and cervical spine stabilized. In cases of rectification of the lumbar lordosis, the lumbar extension and pelvic anteversion movement were performed, keeping the thoracic and cervical spine stabilized. For lumbar hyperlordosis, lumbar flexion and the pelvic retroversion movement were performed with the aid of the deep abdominal muscles, keeping the thoracic and cervical spine stabilized
- *Breathing:* emphasis on inspiration with expansion of the ribs on the concave side of the curve, associated with expiration with emphasis on closing the ribs on the convex side of the scoliotic curvature.
- In all specific exercises of the programme called S4D, the participant was requested to maintain self-correction during the exercises for rotation, sagittal plane, and stabilization or mobilization/stretching training. For final progression training, all patients performed exercises for motor control, balance, function, and dual-task exercises (25).

### Implementation outcomes

The implementation outcomes were used according to the taxonomy of implementation outcomes proposed by Proctor et al. (27). From this taxonomy, the following outcomes were adopted: acceptability, appropriateness, and feasibility, using the Acceptability of Intervention Measure (AIM), Intervention Appropriateness Measure (IAM), and Feasibility of Intervention Measure (FIM) (28). These measures were administered to the physiotherapists and adolescents to determine the extent to which they consider the intervention acceptable, appropriate, and feasible. Each measure contains 4 questions with 5 answer possibilities (1: completely disagree; 2: disagree; 3: neither agree nor disagree; 4: agree; 5: completely agree). The total score ranges from 12 to 65 points and higher scores indicate greater acceptability, appropriateness, and feasibility.

### Statistical analysis

Calculation of the sample size of 66 patients was conducted based on the mean of the pre-intervention Cobb angle, using G-Power 3.0 software. A moderate effect size (f = 0.25), an 80% power, and a 5% significance level were used in the calculation. The normality of the data was verified using the Shapiro–Wilk test. The anthropometric variables and radiographic measurements were compared pre- and post-intervention and between treatments (post-intervention) using Student’s *t*-test. To assess the intra-examiner reliability of the radiographic measurements, the intraclass correlation coefficient (ICC) was used. To calculate the ICC equation type (1, 1) for intra-examiner analysis, measurements were made 1 week apart by the same examiner. The ICC was considered excellent if greater than 0.75, moderate between 0.74 and 0.40, and poor if less than 0.39. To calculate the effect size, Cohen’s d was used, for which the values 0.2, 0.5 and 0.8 were considered to be small, medium, and large effect sizes, respectively. A significance level of 5% for all tests was considered significant. The data were analysed using SPSS version 20.0 (SPSS Inc., Chicago, IL, USA).

## RESULTS

**Fig. 2:**
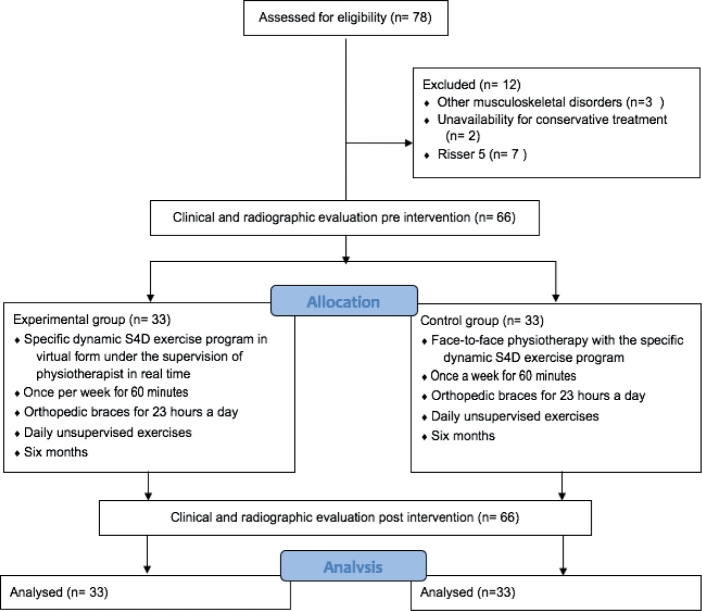
Flow diagram of specific exercises delivered by telerehabilitation or traditional in-person rehabilitation on the progression of scoliotic curvature in adolescents with idiopathic scoliosis (AIS), by the Standard Protocol Items: Recommendations for Interventional Trials (21, 25).

Participants were recruited between January and December 2020. A total of 72 AIS were recruited, 4 of whom did not meet the eligibility criteria and 2 did not complete 6 months of treatment (Fig. 2). Thus, 66 patients were included in the study, 33 for the traditional rehabilitation programme group (boys = 32; girls = 1; mean Risser score = 1.7) and 33 for telerehabilitation (boys = 30; girls = 3; mean Risser score = 2.0) (Fig. 2). The participants were comparable in age, height, body mass, and BMI, and showed no significant differences between groups (Table I). All were right-limb dominant. Inter-observer reliability was high for the spine parameters: Cobb angles (ICC = 0.90); Thoracic Cobb (ICC = 0.89); Lumbar Cobb (ICC = 0.93).

**Table I:** Comparison of anthropometric aspects between groups: traditional rehabilitation programme (in-person) (GP with specific exercises of the programme) and by telerehabilitation (online) of adolescents with idiopathic scoliosis (AIS)

Variables	Telerehabilitation group (mean ± SD)	Traditional in-person rehabilitation group (mean ± SD)	*p*-value
Age (years)	13.4 ± 1.3	14.2 ± 1.3	0.709
Weight (kg)	46.0 ± 8.6	50.1 ± 7.4	0.239
Height (m)	1.5 ± 0.7	1.6 ± 0.7	0.423
BMI (kg/cm^2^)	18.2 ± 3.6	19.6 ± 2.3	0.350

SD: standard deviation.

**Fig. 3:**
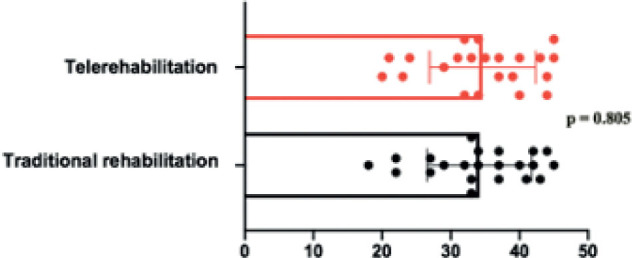
Comparison of the Cobb angle of the main scoliotic curvature after six months of therapeutic intervention by traditional rehabilitation and telerehabilitation in adolescents with idiopathic scoliosis (AIS).

Overall, both groups improved from baseline after 6 months of the intervention, with no differences when compared between treatments (Fig. 3). The variables related to the Nash Moe and Cobb angle in thoracic and lumbar regions did not differ post-intervention, or between groups (Table II). Both groups of patients reported good acceptance and found the intervention appropriate (Table III). Acceptability among the physiotherapists responsible for the treatment with the exercises was also high for both groups (Table IV).

**Table II:** Comparison of clinical and radiographic variables between groups: traditional rehabilitation programme (in-person) and by telerehabilitation (online), pre- and post-6 months after the end of the intervention of the intervention programme

Outcomes	Telerehabilitation group					Traditional in-person rehabilitation group				
	Pre-intervention	Post-6 months after the end of the intervention	d**	MD	*p*-value*	Pre- intervention	Post-6 months after the end of the intervention	d**	MD	*p*-value*
Cobb angle thoracic (°)	35.0 ± 8.7	32.3 ± 9.9	0.28	2.7	0.354	31.4 ± 8.4	28.5 ± 9.7	0.31	2.9	0.305
Cobb angle lumbar (°)	28.6 ± 12.0	28.1 ± 11.0	0.04	0.5	0.899	36.4 ± 9.8	31.2 ± 9.7	0.49	5.2	0.186
Angle Cobb – main scoliotic curvature (°)	37.0 ± 6.8	34.6 ± 7.7	0.33	2.4	0.027*	39.0 ± 5.4	34.1 ± 7.6	0.74	4.9	0.019*
Nash and Moe thoracic (score)	1.2 ± 0.7	1.3 ± 0.6	0.15	0.1	0.675	1.4 ± 0.5	1.5 ± 0.6	0.18	0.1	0.997
Nash and Moe lumbar (score)	1.5 ± 0.7	1.2 ± 0.7	0.42	0.3	0.344	1.8 ± 0.6	1.6 ± 0.6	0.33	0.2	0.989

* Student’s *t*-test, dependent, significant differences *p*<0.05.

** Cohen’s d test to verify the effect of the intervention.

MD: mean difference; Pre-intervention; Post-6 months after the end of the intervention.

**Table III:** Description of the implementation outcomes of acceptability, appropriateness, and feasibility by in-person and telerehabilitation groups

	Traditional in-person rehabilitation group (*n* = 33)	Telerehabilita-tion group (*n* = 33)
AIM, mean (SD)		
The intervention meets my approval.	4.82 (0.39)	4.61 (0.50)
The intervention is appealing to me.	4.58 (0.50)	4.03 (0.68)
I like the intervention.	3.48 (0.76)	3.70 (0.73)
I welcome the intervention.	4.42 (0.56)	4.30 (0.72)
Total score, mean (SD)	**4.33 (0.37)**	**4.16 (0.48)**
IAM, mean (SD)		
The intervention seems fitting.	4.79 (0.42)	4.67 (0.48)
The intervention seems suitable.	4.79 (0.42)	4.58 (0.50)
The intervention seems applicable.	4.61 (0.50)	4.27 (0.72)
The intervention seems like a good match.	4.67 (0.48)	4.61 (0.50)
Total score mean (SD)	**4.71 (0.39)**	**4.53 (0.46)**
FIM, mean (SD)		
The intervention seems implementable.	4.36 (0.60)	4.03 (1.07)
The intervention seems possible.	4.42 (0.61)	4.42 (0.66)
The intervention seems doable.	4.73 (0.45)	4.36 (0.74)
The intervention seems easy to use.	3.82 (0.53)	4.21 (0.82)
Total score mean (SD)	**4.46 (0.34)**	**4.32 (0.46)**

AIM: Acceptability of Intervention Measure; IAM: Intervention Appropriateness Measure; SD: standard deviation; FIM: Feasibility of Intervention Measure.

**Table IV:** Description of the implementation outcomes of acceptability, appropriateness, and feasibility of telerehabilitation by physiotherapists

	Physiotherapists delivering telerehabilitation (*n* = 4)
AIM, mean (SD)	
The intervention meets my approval.	5.0 (0)
The intervention is appealing to me.	4.50 (0.58)
I like the intervention.	3.80 (0.5)
I welcome the intervention.	4.75 (0.5)
Total score, mean (SD)	**4.50 (0.20)**
IAM, mean (SD)	
The intervention seems fitting.	4.25 (0.5)
The intervention seems suitable.	3.75 (0.5)
The intervention seems applicable.	4.5 (0.58)
The intervention seems like a good match.	4.75 (0.50)
Total score, mean (SD)	**4.31 (0.31)**
FIM, mean (SD)	
The intervention seems implementable.	4.5 (0.58)
The intervention seems possible.	4.5 (0.58)
The intervention seems doable.	4.5 (0.58)
The intervention seems easy to use.	4.25 (0.50)
Total score, mean (SD)	**4.44 (0.13)**

AIM: Acceptability of Intervention Measure; IAM: Intervention Appropriateness Measure; SD: standard deviation; FIM: Feasibility of Intervention Measure.

## DISCUSSION

The aims of this study were of this study was to verify and compare the effects of a traditional rehabilitation programme (in-person) and telerehabilitation (online) on the progression of scoliotic curvature in AIS during the COVID-19 pandemic, which resulted in social isolation, making it necessary to find treatment strategies for AIS. The main results of this study showed that traditional rehabilitation and telerehabilitation resulted in a reduction in Cobb angle in the main scoliotic curvature after 6 months of intervention, with a greater effect size for traditional rehabilitation, and that there was good acceptance or the rehabilitation programme among patients and physiotherapists. However, there were no changes in the radiographic aspects of the Cobb angle and thoracic and lumbar and Nash and Moe score pre- and post-6 months of intervention.

The rehabilitation of individuals with idiopathic scoliosis to control and reduce the scoliotic curve has already shown beneficial results through traditional rehabilitation programmes delivered in an in-person individualized manner (21, 29–31). The aims of this study was to verify the effects of telerehabilitation (online) individually, in the current context of the COVID-19 pandemic, as there are no studies evaluating the effects of telerehabilitation in the conservative treatment of scoliosis; however, the efficacy of telerehabilitation has been well-reported for other musculoskeletal disorders, such as following total arthroplasty (e.g. shoulder, knee, hip) and upper limb interventions (e.g. proximal humerus fractures, carpal tunnel release surgery, rotator cuff tear) (32–36). In this context, the findings are promising, as the outcomes commonly considered in postsurgical physical therapy (e.g. reduction in pain intensity and improvements in range of motion, muscle strength, functional activities, and disability) are similar or even superior in comparison with face-to-face usual care - traditional rehabilitation (36).

Traditional rehabilitation is highly effective in reducing and minimizing the progression of scoliosis (37), but, as shown in the current study, telerehabilitation appears to be a promising treatment alternative for patients with AIS, which has benefits, such as ease of access to specialized treatment for those living in more remote areas, and maintenance of social isolation in times of pandemic for infection control. Another benefit of telerehabilitation is the comfort of the patient, who receives the intervention treatment continuously at home through video-calling, with savings in indirect expenses related to transport to a specialized treatment centre (35). Thus, telerehabilitation proved to be effective in the treatment of scoliosis, with the potential to be a safe and effective alternative for AIS assistance.

The main goals of conservative treatment with specific physiotherapy exercises (rehabilitation programme) in AIS are to interrupt or reduce progression of curvature in the skeletal maturity phase, prevent or treat respiratory dysfunction and back pain, and improve aesthetics through postural correction (20). In patients with curvature from 20° to 45°, the performance of a rehabilitation programme, together with wearing a brace for more than 20 h per day, is recommended (20, 21). In the current study, intervention using a traditional rehabilitation programme allowed reduction and control of the main scoliotic curve in AIS, showing more effective than of telerehabilitation. However, the rehabilitation in a clinical setting from a distance using synchronous telerehabilitation; i.e. via a video call with supervision from a physiotherapist, enabled the patient to self-correct during the execution of the exercises, demonstrating a high level of interaction of the AIS participants with the technological resources. Thus, preliminary evidence suggests adopting telerehabilitation in substitution of face-to-face interventions for reducing pain and improving physical function, daily life activities, and quality of life in patients affected by musculoskeletal disorders (38). A strength of the current study was that adolescents with AIS, from both intervention groups, received educational guidance via videos with the exercises proposed provided monthly, which reduced the chances of the exercises not being performed due to lack of understanding.

With the outbreak of COVID-19, telerehabilitation became an important option for physiotherapists to continue treating patients (36). Despite the effectiveness of telerehabilitation for many musculoskeletal conditions, it is important to highlight that the processes of implementation of evidence-based interventions are often regionalized by the cultural diversity and characteristics of a population with scoliosis or low-back pain (25, 37, 38). Thus, it is essential to verify the outcomes of the implementation of telerehabilitation in individuals with AIS. In this context, the current study showed good acceptability, appropriateness, and feasibility of treatment based on telerehabilitation.

Although traditional rehabilitation (in-person physiotherapy) provided a better reduction in the Cobb angle in AIS participants, telerehabilitation was the only treatment option for patients to be able to continue their treatment during the COVID-19 pandemic, and proved to be effective in reducing and preventing the progression of the disease. The American Physical Therapy Association (APTA) supports the use of telehealth to help mitigate rising healthcare costs, address disparity in access to services, and target healthcare in regions of the country where there is a shortage of treatment for adolescent patients with scoliosis (39). Virtual physical therapy has been shown to be an effective service for provision through telerehabilitation, showing improvements in function as an additional mode of evaluation and treatment (39, 40).

The results of the current study corroborate those of other studies of musculoskeletal disorders that reported good overall satisfaction with telerehabilitation (33). Satisfaction is one of the variables that can be used to analyse the appropriateness of implementation of a service (27). Telerehabilitation and virtual physical therapy are innovative and cost-effective ways to provide the best rehabilitative services to patients in their homes (40). The current study data suggest that individualized and personalized interventions can be approved regardless of the delivered care provided (13), and telerehabilitation is an option for effective delivery of care that can be offered to users with AIS.

According to Shah et al. (41), telerehabilitation achieved a significantly better reduction in pain and disability among patients with spine pain than in-clinic rehabilitation. These encouraging results during the COVID-19 pandemic indicate the need to further explore and test the efficacy and wider application of telerehabilitation for treating spine pain. In contrast, in the current study, traditional rehabilitation proved to be more effective in reducing the Cobb angle compared with telerehabilitation. However, telerehabilitation also promoted progression control and reduction of the Cobb angle in patients. An important explanation is the care in the application of telerehabilitation to treat patients with spine disorders within the framework of ethical principles (42) is essential for the protection of patient privacy. Ethical principles in telerehabilitation include universal accessibility, patient-informed consent, respect for privacy, professional confidentiality, and patient data safety (42, 43). The authors and physical therapists who delivered telerehabilitation in the current study aimed to ensure that the highest ethical standards were maintained. All therapists involved in telerehabilitation were trained in its ethical aspects, which included taking informed consent, ensuring professional patient-caregiver confidentiality during all communications, and patient data safety.

A systematic review of qualitative studies explored the acceptance of real-time 1:1 videoconferencing with patients in an orthopaedic setting, and observed that a common theme running through the included studies was that videoconferencing is convenient because access to remote healthcare reduces costs and saves time for the patient (44). Another important point is the behaviour of the therapists to promote a therapeutic relationship with the patient. The systematic review identified that characteristics such as staring at the screen (rather than moving gaze from camera to screen), listening without interruption, and individually tailoring exercises to the patient’s individual needs facilitated relationship-building (44).

In telerehabilitation, exercises must be adjusted by the physiotherapists to suit the patient’s home environment and, for treatment of scoliosis, it can be considered as a differential in the treatment because of the need for the physiotherapist to adapt the exercises to the daily functional activities of the patient. In addition, the therapist-patient interaction tends to be more intense than in other healthcare professions, due to the nature of the rehabilitation treatment and extended time of assistance (39, 45). Thus, it is necessary to establish satisfaction with, and acceptability of, the healthcare used in order to offer the treatment to other patients and prioritize patient-centred care, providing different options for the delivery of evidence-based interventions for AIS.

### Limitations

In the current study, there was a good satisfaction of the physiotherapists with the telerehabilitation modality. The physiotherapists seem to agree that tele-healthcare is similar to in-person care when focused on exercise, education, and activity modification (46, 47), but that the lack of physical contact limits the provision of hands-on passive treatments (46). A limitation of this study is the non-differentiation of the different types of scoliotic curvature in both treatment protocols: telerehabilitation and traditional rehabilitation for AIS. This understanding can better establish the clinical response of AIS in telerehabilitation care. Future studies should this issue into account to enable improved planning of the rehabilitation programme for telerehabilitation in spine disorders, especially scoliosis.

### Conclusion

A rehabilitation programme for AIS, delivered via telerehabilitation during the COVID-19 pandemic, was encouraging for future use due to the positive effect on reducing the Cobb angle, preventing progression of scoliosis. Traditional rehabilitation programmes (delivered in-person) also showed a reduction in the Cobb angle in AIS. In addition, there was good acceptability of telerehabilitation among patients and physiotherapists.
